# Mass rabies exposure of veterinary health care workers in Germany: Management, immune response, and tolerability of post exposure-prophylaxis

**DOI:** 10.1016/j.onehlt.2025.100978

**Published:** 2025-01-19

**Authors:** Lennart Lemmermann, Jonathan Remppis, Sabine Bélard, Florian Steiner

**Affiliations:** a Institute of Tropical Medicine, University of Tübingen, Tübingen, Germany; b Department of Dermatology, Venereology and Allergology, Bundeswehrkrankenhaus, Hamburg, Germany; c German Center for Infection Research (DZIF), Partner Site Tübingen, Tübingen, Germany; d Department of Pediatric Neurology, General Pediatrics, Diabetology, Endocrinology and Social Pediatrics, University Children's Hospital Tübingen, Tübingen, Germany; e Praxis Dr. Steiner, Tarmstedt, Germany

**Keywords:** Rabies, Mass exposure, Post-exposure prophylaxis, Illegal pet trade, Germany, Vaccination strategies

## Abstract

**Background:**

Rabies is a fatal zoonotic disease caused by *Rabies lyssavirus*, primarily transmitted through dog bites. In 2008, Germany was declared free from terrestrial rabies by the WOAH. However, illegal pet imports can still lead to rabies exposure, as seen in the 2021 case of a rabid puppy illegally imported to Germany, resulting in a mass exposure incident.

**Methods:**

This retrospective study assessed the post-exposure management of 39 veterinary clinic staff exposed to the rabid puppy, focusing on the practicability, immunogenicity, and tolerability of post-exposure prophylaxis (PEP). Data was collected from patient records and for follow-up by phone using standardized case record forms, entered into an Excel database, and analyzed descriptively. Ethics approval was obtained from the Ethics Committee of the University of Tübingen (8452022BO2).

**Results:**

Exposed individuals received varying PEP regimens based on vaccination status and exposure level, including possible aerosol exposure. Most followed the Essen-Scheme, with some receiving human rabies immune globulin (hRIG). All developed protective antibody titers, and adverse reactions were generally mild.

**Conclusion:**

This case highlights challenges in rabies mass exposure management and emphasizes needs for continued preparedness, resource allocation and education about rabies, even in regions considered rabies-free.

## 1 Introduction

Rabies, a zoonotic viral disease, remains a significant global public health concern, despite being preventable through pre- and post-exposure prophylaxis (PrEP and PEP) [1,2]. The disease is almost invariably fatal once clinical symptoms appear. However, the development of disease following exposure can be effectively halted if appropriate measures are taken in time after exposure [3].

In Germany, as in western and central parts of Europe, Australia, New Zealand and Japan, terrestrial rabies in wild and domestic animals has been eliminated by systematic control measures, particularly immunization of foxes and domestic animals. Since 2008, Germany has been declared free of terrestrial rabies according to the World Organization for Animal Health (WOAH) [4]. However, the threat of rabies persists, especially considering the ease of international animal transport and increasing illegal pet import as these animals may not be vaccinated against rabies as per regulations or may enter the country being already infected with rabies and putting animals or human contact persons at risk [5].

The management of rabies exposure is multifaceted, involving immediate wound care, risk assessment, procurement and administration of PEP, monitoring for adverse reactions and vaccine efficacy, as well as education of exposed people [3]. The guidelines provided by institutions such as the Robert Koch Institute (RKI) in Germany [6] play a crucial role in guiding healthcare professionals through this process. However, real-world scenarios, particularly those involving mass exposure events and uncommon exposure risks, can present unique challenges that extend beyond standard protocols.

This retrospective study aimed to delineate the practical aspects and challenges of managing a rabies mass exposure event to a post-mortem confirmed rabid domestic dog that was cared for in a veterinary clinic setting in Germany in 2021 [7] and to evaluate the immunogenicity and tolerability of PEP. By highlighting the lessons learned from this unique event, this study contributes to the broader understanding of mass rabies exposure management and identifies key areas for improvement in preparedness and response.

## 2 Methods

This was a retrospective study evaluating patient files documenting post-exposure assessment and care of veterinary clinic staff, exposed to a rabid puppy in 2021 in Germany, and uncommonly primarily managed by a family doctor, due to challenges in obtaining a timely response from the seven responsible district health authorities. Post-exposure care comprised assessment of exposure level as well as pre-exposure vaccination status, passive and active immunizations, post-immunization assessment of antibody levels against rabies vaccine, and assessment of adverse reactions to vaccination. Data collection was performed by standardized data extraction from the family doctor's documentation in case files using predefined paper-based case record forms (CRF). For follow-up data, patients were contacted by phone for assessment of survival and recording of complications. The recorded data comprised clinical data, immunization data, vaccine response data, and vaccine tolerability data. The CRFs were subsequently digitized by entering the data into a pre-configured Excel database. Data was analyzed in a descriptive way. Ethics approval was obtained from the Ethics Committee of the University of Tübingen (8452022BO2).

## 3 Results

### 3.1 Index case

**Fig. 1:**
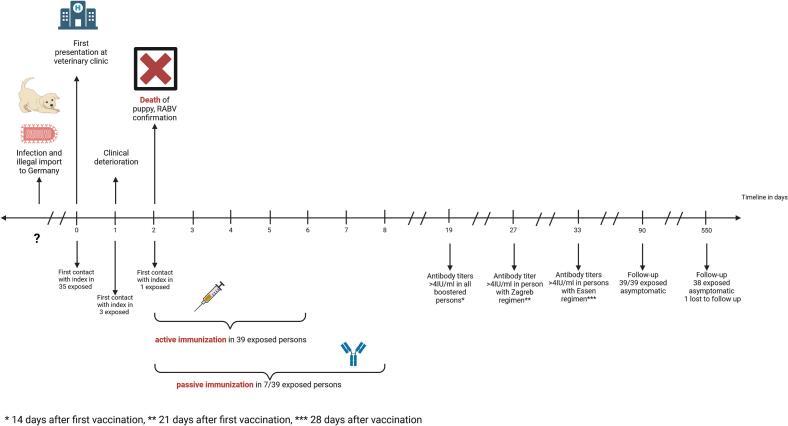
Timeline of the rabies mass exposure event and post-exposure prophylaxis management.

A Kangal puppy with an estimated age of 8 weeks was presented sick, soaked and hypothermic, to a veterinary clinic by his owners. According to the owners, the puppy originated from an eastern Mediterranean region and had been in Germany for 2.5 weeks. A significant deterioration of the puppy's general condition had occurred during the preceeding days. The owners stated that the puppy had stopped drinking and that, therefore, they had (inappropriately) pushed hydration by temporarily holding the puppy under water to support fluid intake. Initial veterinary management comprised a condition check, as well as drying and warming of the puppy with a blow-dryer for approximately 30 min at the surgery preparation area, a pass-through room were other animals were also being cared for simultanously. Thereafter, the puppy was transferred to the recovery unit. However, the next day the puppy's general condition deteriorated; the puppy showed lethargic, a drop in temperature to 34 °C, and rotatory nystagmus. The puppy died two days after admission. The clinical course and suspected illegal importation raised the suspicion of rabies. Post-mortem investigations confirmed rabies by direct fluorescent antibody testing (dFA) followed by RT-qPCR on the day the puppy passed away. Subsequent sequencing showed a 99.5 % match with a RABV sample from a Turkish fox diagnosed with rabies in 2001 [7]. The timeline of events is depicted in Fig. 1.

### 3.2 Exposure cluster and management

This study evaluated the post-exposure management of a total of 39 employees of the veterinary clinic cared for by a single local primary care physician. PEP management of further exposed people (including the puppy's owners) that was provided outside the family doctor's practice, is not reported here. The 39 employeses of the veterinary clinic included veterinarians, veterinary assistants, receptionists, and an animal keeper; all of them experienced some type of exposure to the rabid puppy during its inpatient care (Table 1). The cohort of exposed individuals comprised 33 women and 6 men with a median age of 27 years (IQR 24;35). None of the exposed had an immunocompromising condition.

**Table 1:** Initial and follow-up exposure grades, pre-immunization status, and post-exposure-prophylaxis regimen.

Initial exposure grade [6]	Type and location of exposure (final)	Potential aerosol exposure, direct/indirect^a^ (duration of exposure (min))	Corrected/final exposure grade [6]	Pre-immunization (year (n prior doses))	Day of PEP initiation^b^, PEP-Scheeme	Day(s) of hRIG, total dose (dose per kgBW), administration site^c^
3	licking injured skin (hands) and mucous membrane	direct (5)	3	no	D2, Essen	D8 1800 IU (20 IU/KgBW) palms, upper arms
3	licking injured skin (hands)	indirect (5)	3	no	D2, Essen	D8 1200 IU (20 IU/KgBW) palms, upper arm
3	nibbling/licking injured skin (hands, arm)	direct (30–45)	3	no	D2, Essen	D8 1320 IU (20 IU/KgBW) palms, right upper arm
3	bitten/nibbling/licking intact skin (hands)	no	3	no	D2, Essen	D6 750 IU (10,7 IU/KgBW, intragluteal D8 660 IU (total 20 IU/KgBW), left palm
3	bitten/nibbling/licking injured skin (right hand)	no	3	no	D1, Essen	D1 1500 IU (17,2 IU/KgBW), intragluteal D6 1740 IU (20 IU/KgBW) right palm, right upper arm
3	bitten (décolleté)/licking injured skin (hands)	direct (30–45)	3	no	D2, Essen	D6 750 IU (12,5 IU/KgBW) intragluteal D7 1200 IU (20 IU/KgBW) décolleté, right upper arm
3	touch of oral mucosa, nibbling/licking injured skin (hands)	direct (30–45)	3	2002 (3) (initially unknown)	D2, Essen^d^	D2 1500 IU (25 IU/KgBW), thighs
2	touch with intact skin (hands)	no	1	2016 (3)	D3, Booster	no
2	touch with intact skin (hands)	no	1	2019 (3)	D4, Booster	no
2	licking/nibbling/scratching non-intact skin (hands/arms)	no	2	no	D2, Essen	no
2	licking non-intact skin (hands/arms)	indirect (10)	2	no	D2, Essen	no
2	licking non-intact skin (hands)	no	2	no	D3, Essen	no
2	touch with injured skin	indirect (30–45)	2	no	D2, Essen	no
2	touch with intact skin (hands)	indirect (10)	1	no	D2, Essen,	no
2	touch with intact skin (hands)	no	1	no	D2, Essen	no
2	touch with intact skin (hands)	indirect (30–45)	1	no	D2, Essen	no
2	touch with intact skin (hands)	no	1	no	D2, Essen	no
2	licking intact skin (hands)	direct (30–45)	1	no	D3, Essen	no
2	licking intact skin (hands)	direct (30–45)	1	no	D2, Essen	no
2	touch with intact skin (hands)	no	1	no	D2, Essen	no
2	touch with intact skin (hands)	no	1	no	D1, Essen	no
2	touch and inventory cleaning with intact skin (hands)	no	1	no	D2, Essen	no
2	touch with intact skin (hands)	no	1	no	D2, Essen	no
2	touch with gloves (hands)	no	1	no	D0, Essen	no
2	licking intact skin (hands)	no	1	no	D2, Essen	no
unclear	potential aerosol exposure	indirect (5)	unclear	2011 (3)	D2, Booster	no
unclear	potential aerosol exposure	indirect (1)	unclear	2016 (3)	D5, Booster	no
unclear	potential aerosol exposure	indirect (5)	unclear	2008 (3)	D2, Booster	no
unclear	potential aerosol exposure	indirect (30–45)	unclear	2008 (3) 2011 (1) 2013 (1)	D3, Booster	no
unclear	potential aerosol exposure	indirect (1)	unclear	2005 (2)	D5, Booster	no
unclear	potential aerosol exposure	direct (30–45)	unclear	no	D4, Essen	no
unclear	potential aerosol exposure	indirect (5)	unclear	no	D2, Essen	no
unclear	potential aerosol exposure	indirect (10)	unclear	no	D3, Essen	no
unclear	inventory cleaning with injured skin (hands)	indirect (2)	unclear	no	D2, Essen	no
unclear	potential aerosol exposure	Direct (30–45)	unclear	no	D2, Essen	no
unclear	potential aerosol exposure	Indirect (30–45)	unclear	no	D2, Essen	no
unclear	potential aerosol exposure	indirect (1)	unclear	no	D2, Essen	no
unclear	potential aerosol exposure	direct (5)	unclear	no	D2, Essen	no
unclear	potential aerosol exposure	indirect (10)	unclear	no	D6, Zagreb	No

min: minutes, n: number, PEP: post-exposure prophylaxis, hRIG: human rabies immune globulin; kgBW: kilogram body weight; D: day; IU: international unit;

a Directly involved: present in the immediate proximity and/or actively blow-drying; indirectly involved: present in the same room or passing the room during blow-drying without being in the immediate proximity.

b Active vaccination scheemes: Booster (Days 0, 3), Essen-Scheme (Days 0, 3, 7, 14, 28), Zagreb-Scheme (Days 0, 0, 7, 21).

c Recommended dose according to World Health Organization/Robert Koch Institute: 20 IU/KgBW.

d Terminated after 4th. vaccination due to new information on previous immunization.

Mass exposure management for employees of the veterinary clinic was initiated on the day of the puppy's rabies confirmation through the veterinary clinic, the on-call statutory health insurance physician, and primarily the family doctor's practice, located in the vincinity of the veterinary clinic. Of note, rabies confirmation results reached the veterinary clinic on Friday evening and initial exposure management had to be dealt with outside regular working hours of the responsible health authorities. First, exposure risk stratification according to national Robert Koch Institute's (RKI) recommendations [6] was partly performed via phone consultation with exposed. In summary, grading was done into three expose grades based on the type of contact with the rabid puppy. Exposure Grade I (low risk): Contact with the rabid puppy that did not involve a bite, scratch, or saliva exposure to broken skin or mucous membranes. Exposure Grade II (moderate risk): Minor bites or scratches from the rabid animal or licking of broken skin or mucous membranes. Exposure Grade III (high risk): Severe bites or scratches, or direct exposure to saliva or nervous tissue (e.g., through open wounds or mucous membranes). Due to repeated exposures over up to three days prior assessment, exact determination of unambigous exposure grades proved challenging in many cases, particularly taking into account the extremely unlikely but possible risk exposure via aerosol [8]. Initial exposure levels were grade 3 (bite or scratch wounds, contact of mucous membranes or wounds with saliva) in 7 (18 %) exposed and grade 2 (non-bleeding superficial scratches or skin abrasios, licking or nibbling non-intact skin) in 18 (46 %) exposed. Potential aerosol exposure was not assigned to any of the three standard exposure grading categories but was instead classified as an unclear exposure. Fourteen (36 %) individuals with exclusive possible exposure to potentially infectious aerosols for up to 30 min arising from blow-drying the puppy, were classified with unclear exposure. In some cases, the exposure grade was corrected during follow-up, as the initial assessment, often conducted over the phone, was later refined through an in-person evaluation involving a physical examination. Additionally, some individuals were highly anxious during the initial assessment, leading to uncertainty about possible skin lesions and, consequently, a more cautious, higher exposure classification. Exposure details and initial as well as final grading are presented in Table 1.

Emergency access to rabies vaccines and human rabies immune globulin (hRIG) was a key first measure but proved difficult due to official local vaccines storages being insuffiently stocked for mass exposure events. The clarity regarding responsibility for costs was also lacking, resulting in the local pharmacy and the family doctor having to bear the upfront cost of several thousand euros for vaccines, with no assurance of reimbursement. Needed vaccines were compiled by the veterinary clinic, the stock of the family doctor's practice, and local pharmacies. The Essen scheme was favored over the Zagreb scheme because it requires only a single dose on Day 0, whereas the Zagreb scheme involves two doses on Day 0. Given the limited availability of vaccine doses, the Essen scheme with only a single dose on Day 0 ensured that all exposed could timely receive a first vaccine dose and allowed time for further vaccine procurement over the weekend. The time interval between first exposure and initiation of PEP ranged from 1 to 7 days post exposure. All grade 3 exposed individuals (7/39, 18 %) received hRIG in addition to the Essen-Scheme (five single doses active vaccinations with Rabipur® at intervals of 0,3,7,14,28 days). HRIG was administered within seven days of first active immunization; hRIG regimen deviated from recommendations in 4/7 exposed (Table 1). Exposed individuals with previous complete anti-rabies immunization (7/39, 18 %) were given a two-dose booster (days 0,3). Of 32 exposed individuals without previous anti-rabies immunization 31 (97 %) received the Essen-Scheme and 1 (3 %) received the Zagreb-Scheme (four doses active vaccination with Rabipur® at intervals of 0, 0,7,21 days). Overall, a total of 173 vaccine doses were administered and PEP regimen were completed in all but one exposed (early termination of PEP because of clarification on the immune status in the meantime). Allmost all vaccines were administered at the family doctor's practice; hRIG was administered at emergency departments of hospitals close by.

Active and passive immunizations were generally well tolerated; reported adverse reactions are summarized in Table 2. Antirabies antibody determination was performed once for each patient on day 14 (*n* = 7; booster), day 21 (*n* = 1; Zagreb-Scheme), or day 28 (*n* = 31; Essen-Scheme) after first active vaccination. Post-PEP antirabies antibody titers (neutralization test) were > 4 IU/ml in all individuals. All exposed were asymptomatic at follow-up 90 days after exposure; at follow-up 18 months after exposure 38/39 exposed were asymptomatic, one individual was lost to follow-up. Time flow of the puppy's clinical course and mass exposure interventions is presented in Fig. 1.

**Table 2:** Adverse reaction after active and passive immunization; numbers indicate number and proportion of patients with adverse reaction; shading illustrates the proportion of patients with adverse reaction (the darker the higher the proportion).

Adverse reactions after active immunization																			
	PEP Essen-Scheme (*n* = 24) n (%)						PEP Zagreb-Scheme (n = 1) n (%)				Booster (n = 7) n (%)			PEP Essen-Scheme plus hRIG (n = 7) n (%)					
	D0 (n = 24)	D3 (n = 24)	D7 (n = 24)	D14 (n = 24)	D28 (n = 24)	Total doses (*n* = 120)	D0 (n = 2)	D7 (n = 1)	D21 (n = 1)	Total doses (*n* = 4)	D0 (*n* = 7)	D3 (*n* = 7)	Total doses (*n* = 14)	D0 (n = 7)	D3 (n = 7)	D7 (n = 7)	D14 (n = 7)	D28 (*n* = 6)	Total doses (*n* = 34)
Local pain	6 (25)	9 (38)	7 (29)	7 (29)	4 (17)	33 (28)	0	0	0	0	3 (43)	2 (29)	5 (36)	3 (43)	3 (43)	4 (57)	3 (43)	2 (33)	15 (44)
Malaise	5 (21)	6 (25)	6 (25)	6 (25)	4 (17)	27 (23)	2 (100)	1 (100)	0	3 (75)	4 (57)	2 (29)	6 (43)	2 (29)	2 (29)	3 (43)	3 (43)	1 (17)	11 (32)
Cephalgia	6 (25)	5 (21)	3 (13)	4 (17)	2 (8)	20 (17)	2 (100)	1 (100)	0	3 (75)	2 (29)	1 (14)	3 (21)	4 (57)	4 (57)	5 (71)	3 (43)	2 (33)	18 (53)
Fever	1 (4)	0	0	0	0	1 (1)	0	0	0	0	0	0	0	0	0	0	0	0	0
Nausea	2 (8)	1 (4)	2 (8)	1 (4)	1 (4)	7 (6)	2 (100)	0	0	1 (20)	0	0	0	0	0	0	0	0	0
Allergic reaction	0	0	0	1″ (4)	0	1 (1)	0	0	0	0	0	0	0	0	0	0	0	0	0
Other	2^⁎^ (8)	1^⁎^ (4)	3^⁎x# (^13)	0	0	6 (5)	0	0	0	0	0	0	0	0	0	1^‘^(14)	0	0	1 (3)


Adverse reactions after passive immunization
Case 1: fever, prolonged pain (3 weeks); Case 2: malaise, headache, nausea; Case 3: malaise (1 week), cephalgia, muscle pain; Case 4: numbness; Case 5: swelling, numbness; Case 6: malaise; Case 7: pain.

PEP: post-exposure prophylaxis, hRIG: human rabies immune globulin; D0: dose on day 0, D3: dose on day 3, D7: dose on day 7, D14: dose on day 14, D21: dose on day 21, D28: dose on day 28, n: number.

“Grade 1 according to the WAO evaluation system for systemic allergic reactions [9], * vertigo; x neck pain; # lymph node swelling; ‘syncope (vasovagal).

## 4 Discussion

The occurence of a domestic rabies case in Germany in 2021, a country considered free of terrestrial rabies since 2008, marked a significant exception from the country's official status. A single importation of a rabid dog, with appropriate contact tracing, would not impact the country's rabies status, as the virus would need to spread substantially within the wildlife or feral dog population to affect national status. However, this incident - stemming from an illegally imported dog - highlights the specific rabies risks tied to illegal animal trade, posing health risks not only for pet owners but also for veterinary care workers. Furthermore, it raises public health concerns due to the challenges in tracing both the index case and any potential secondary cases. However, imported canine rabies is not solely associated with illegal import as illustrated by two cases of rabid dogs imported to the United States and Canada from Azerbaijan and Iran. Both dogs arrived with rabies vaccination certificates and were part of declared animal rescue shipments [10,11]. In one case rabies vaccination failure was assumed due to underdosed vaccination rather than fraudulent or incomplete paperwork, illustrating that rabies vaccination certificates alone will not ensure that dogs from high-risk countries for rabies have adequate protection against rabies [10].

Mass rabies exposures in countries free of terrestrial rabies have been described as mass bat exposures [12] or exposure of health care workers to human rabies cases [13]. To the best of our knowledge no previous mass exposure event due to an illegally imported pet to a country free of terrestrial rabies has been reported. The exposure of 39 veterinary clinic staff members to a rabid puppy in Germany represents a significant mass exposure event requiring immediate PEP for a considerable number of exposed in a country where PEP is mostly administered only for single exposed individuals after travel related exposure.

This incident highlights the complexity of managing rabies exposure in real-world settings, particularly in mass exposure events. Mass exposure events require immediate availability of larger amounts of vaccines and immune globulins that may be difficult to obtain, particularly on weekends as in this case. During the described event, one of the significant logistical challenges was accessing emergency vaccine stockpiles on a weekend, when many resources were unavailable or difficult to locate. This delay underscores the need for a more robust and accessible system of emergency vaccine stockpiles. To address this, we recommend establishing clearly defined and well-maintained stockpiles that are strategically distributed and available for access outside regular business hours, including weekends and holidays. Publicly accessible, up-to-date information on stockpile locations and vaccine availability would further enhance preparedness. Furthermore, this case demonstrates that timing of the incident may not only compromise vaccine procurement, but also impede reachability of exposed individuals (particuarly in the context of occupational exposure). Another challenge that was also experienced in this case, is the need for a timely initial risk stratification under staff constraints and with incomplete information. From a financial perspective, while eventual reimbursement by the insurance company was uncomplicated, the family doctor initially bore the financial burden of procuring the vaccines. This risk poses a significant barrier to the immediate management of mass exposure events if stockpiles are inaccessible or insufficiently stocked. Furthermore, had the confirmation test for rabies in the puppy turned out negative upon confirmation testing, the family doctor could have been left with unused vaccines and no pathway for reimbursement, further emphasizing the vulnerability of the current system. To address these issues, we propose actionable recommendations, including guarantees of cost coverage by public health authorities during mass exposure events and improved coordination to ensure healthcare providers can access emergency stockpiles without the need for direct procurement.

In this mass exposure event, health care staff additionally needed to timely deal with the particular question of potential rabies transmission via aerosols by blow-drying the soaked rabied puppy. Such a scenario is not explicitly covered in existing guidelines, and in the acute situation risk assessment lies in the reponsibility of the management person or team. The respective health authorities were consulted regarding this specific circumstance, but a definitive answer was pending due to the lack of precedent for such a scenario. To ensure the best possible decision-making, experts from the German Society of Tropical Medicine, Travel Medicine, and Global Health, as well as recognized supranational rabies experts, were contacted. These experts supported the decision to adopt a cautious approach, emphasizing the high-risk nature of rabies and the need to prioritize safety. Aerosol exposure should generally be considered in case of high concentration of virus suspended in the air that could contact a person's mucous membranes or be inhaled, such as in densely populated bat caves [14] or when handling concentrated laboratory samples of the virus [15]. However, there is no documented transmission experience on blow-drying rabid puppies. In the case of this potential aerosol exposure relating to a confirmed rabid animal, the lethal course of rabies justified the assumption of a worst-case scenario. Given that blow-drying typically does not present a splash risk and hot-air drying would reduce the survival of any virus particles present on the dog's surface, do however, make contagious aerosol exposure highly improbable. Although to date only two possible aerosol-based cases of laboratory-acquired rabies infections have been reported [8,18] WHO recommendations state that in case of suspected RABV exposure through aerosols, PEP including hRIG injection can be provided, based on a risk assessment [3]. In summary, national guidelines served as the primary reference for exposure grading. However, given the unique circumstances of this mass exposure event—including limited resources, potential aerosol exposure, heightened psychological fears among exposed individuals, the confirmed rabies infection in the index animal, and the inherently high-risk nature of rabies—a cautious approach was adopted in cases with initial uncertain exposure grade. This precautionary stance was driven by the need to prioritize safety in a situation where uncertainties and logistical challenges were compounded by the scale of the event. Rabies is an almost invariably fatal disease once clinical symptoms appear, and ensuring adequate prophylaxis in potentially exposed individuals is paramount.

To date, there has been no reported transmission to healthcare workers during their care of patients with rabies [16,17]; whether this applies as well to veterinary healthcare workers during their care of animals with rabies is not reported. Given the lack of human-to-human rabies transmission a conservative approach has been suggested for PEP in exposed HCWs [12].

The successful completion of PEP and the subsequent antibody titers above 4 IU/ml [19] among all exposed individuals demonstrates effective mass exposure management and adequate immunogenicity of the administered PEP regimens. Adequate antibody titers were achieved before administration of the last (fifth) vaccine dose, supporting the evidence that a reduced, 4-dose intramuscular vaccine schedule in healthy patients seems non-inferior to the 5-dose schedule [20]. However, well-designed studies with larger samples sizes are needed to confirm the sufficiency of a 4-dose regimen. The absence of occuring rabies disease in the follow-up period might be a testament to the effectiveness of the current PEP regimens; however, it remains unknown whether true rabies transmission occurred and PEP effectively prevented rabies disease. Timely and appropriate administration of PEP is crucial to prevent rabies, possible causes for breakthrough infections, that rarely occur, include errors in the administration of rabies immunoglobulin, delays in seeking health care, and comorbidities or immunosuppression [21].

The well documented good tolerability of PEP in this cohort is in line with the longstanding reported good tolerability of the respective active vaccine used in this PEP management [22] and another mass PEP administration study [16]. Concerns about the general unfavorable tolerability of currently licensed rabies vaccine, still circulating in the community, seem unsubstantiated. In contrast, psychological stress associated with rabies exposure, particularly in non-immunized patients in need for hRIG administration, can be immense. In this cohort, hRIG was not immediately available for all patients in need for hRIG and additionally hRIG administration was not in line with recommendations in all patients; this underscores the need for better preparedness and resource allocation in emergency situations to, first of all, optimize effectiveness of PEP but also reduce the concurrent psychological burden in exposed individuals.

Our study is limited by its retrospective nature and the reliance on case file data, which may not capture all nuances of individual exposures and management decisions. However, the thorough documentation of exposure management in all exposed vetinary staff allowed a unique retrospective evaluation of immunogenicity and tolerability of rabies PEP.

In conclusion, the management of a mass rabies exposure incident, as detailed by this study, highlights critical aspects of PEP management and the challenges faced in real-world scenarios. This event serves as a reminder of the persistent risk of rabies, even in countries where the disease is considered eliminated and underscores the need for improved preparedness. Key measures include improving access to well-stocked vaccine stockpiles and developing clear, standardized guidelines for managing mass exposure and unusual exposure events. Additionally, continuous education of healthcare and veterinary professionals as well as stricter regulation and monitoring of animal trade are essential to mitigate risks of rabies exposure and improve management in case of rabies exposure.

## Statements and declarations

The authors did not receive support from any organization for the submitted work besides support from the Open Access Publication Fund of the University of Tübingen.

## CRediT authorship contribution statement

**Lennart Lemmermann:** Writing – review & editing, Writing – original draft, Formal analysis, Data curation, Conceptualization. **Jonathan Remppis:** Writing – review & editing, Writing – original draft, Supervision, Formal analysis. **Sabine Bélard:** Writing – review & editing, Writing – original draft, Visualization, Validation, Supervision, Resources, Project administration, Methodology, Investigation, Formal analysis, Conceptualization. **Florian Steiner:** Writing – review & editing, Writing – original draft, Supervision, Project administration, Methodology, Investigation, Formal analysis, Data curation, Conceptualization.

## Declaration of competing interest

The authors declare that they have no known competing financial interests or personal relationships that could have appeared to influence the work reported in this paper.

## Data Availability

Data will be made available on request.
